# Supplementary material for: Sentiment analysis of medical record notes for lung cancer patients at the Department of Veterans Affairs
Source: PLoS One. 2023 Jan 25;18(1):e0280931. doi: 10.1371/journal.pone.0280931 (PMC9876289; doi:10.1371/journal.pone.0280931)
Supplement: Attachment — Submitted filename: Response to Reviewers.docx [file pone.0280931.s004.docx]

# Response to Reviewers (PONE-D-22-11193)

Based on comments from the reviewer and the additional requests the manuscript ‘Sentiment analysis of medical record notes for lung cancer patients at the Department of Veterans Affairs.’ has been adjusted. Our response can be found below.

## Additional Requests

1. Please ensure that your manuscript meets PLOS ONE's style requirements, including those for file naming. The PLOS ONE style templates can be found at
   <https://journals.plos.org/plosone/s/file?id=wjVg/PLOSOne_formatting_sample_main_body.pdf> and 
   <https://journals.plos.org/plosone/s/file?id=ba62/PLOSOne_formatting_sample_title_authors_affiliations.pdf>

*File names have now been updated according to the style requirements.*

2. Please include an ethics statement in your Methods section, clarifying whether or not the study was approved by an IRB, and provide the name of the IRB and any approval number.

*The following text has been added to the Methods – Selected Data section:*

*This study was approved by the VA Boston Healthcare System Institutional Review Board (3209), under a waiver of informed consent. Access to raw data from the United States Department of Veterans Affairs corporate data warehouse was granted by the VA Boston Healthcare System Institutional Review Board and the Veterans Health Administration National Data Systems.*

3. Please note that PLOS ONE has specific guidelines on code sharing for submissions in which author-generated code underpins the findings in the manuscript. In these cases, all author-generated code must be made available without restrictions upon publication of the work. Please review our guidelines at <https://journals.plos.org/plosone/s/materials-and-software-sharing#loc-sharing-code> and ensure that your code is shared in a way that follows best practice and facilitates reproducibility and reuse.

*The code has been made available through Github, under the MIT license, link:* [*https://github.com/delbers/SentimentAnalysisOfMedicalRecordNotes*](https://github.com/delbers/SentimentAnalysisOfMedicalRecordNotes)

*The ‘Code Availability’ section has been added to the manuscript.*

4. Please provide additional details regarding participant consent. In the ethics statement in the Methods and online submission information, please ensure that you have specified (1) whether consent was informed and (2) what type you obtained (for instance, written or verbal, and if verbal, how it was documented and witnessed). If your study included minors, state whether you obtained consent from parents or guardians. If the need for consent was waived by the ethics committee, please include this information. If you are reporting a retrospective study of medical records or archived samples, please ensure that you have discussed whether all data were fully anonymized before you accessed them and/or whether the IRB or ethics committee waived the requirement for informed consent. If patients provided informed written consent to have data from their medical records used in research, please include this information.

*Addressed in 2.*

5. We note that you have indicated that data from this study are available upon request. PLOS only allows data to be available upon request if there are legal or ethical restrictions on sharing data publicly. For information on unacceptable data access restrictions, please see <http://journals.plos.org/plosone/s/data-availability#loc-unacceptable-data-access-restrictions>.

*Input data in regard to sentiment scoring and the word scores used and generated through the analysis are made available through Github:*

[*https://github.com/delbers/SentimentAnalysisOfMedicalRecordNotes*](https://github.com/delbers/SentimentAnalysisOfMedicalRecordNotes)

*This has been added to the Data Availability section.*

*There are indeed legal and ethical restrictions in place on sharing the patient data publicly as this is identifiable health record data, including 3.5 million notes. De-identification of clinical notes is a highly complex process and to date there is no automated system approved in the Department of Veterans Affairs to perform this. The current data availability statement reads as follows.*

*‘Patient related and note data cannot be shared publicly because it involves sensitive human subject data.*

*Data may be available for researchers who meet the criteria for access to confidential data after evaluation from VA Research and Development Committees. As a VA national legal policy (VHA Directive 1605.01), VA will only share patient data if there is a fully executed contract in place for the specific project.*

*A common contractual mechanism utilized for this type of sharing is a ‘Cooperative Research and Development’ (CRADA) agreement.*

*These contracts are typically negotiated in collaboration with VA national Office of General Council (OGC) and lawyers from the collaborating institution.*

*These national sharing policies and standards also apply to deidentified data.*

*In addition, if a contract is in place allowing sharing of deidentified data outside of VA, then VA national policy (VHA Directive 1605.01), states that deidentification certification needs to be met by Expert Determination. The expert determination requires independent assessment from an experienced master or PhD in biostatistics, from a third party not involved in the project, and may require outside funding to support. In addition, for an outside entity to preform research on VA patient data, IRB as well as VA Research and Development Committee approval is required for the specific project. Data requests may be sent to: VA Information Resource Center (VIReC) Building 18 Hines VA Hospital (151V) 5000 S. 5th Avenue Hines, IL 60141-3030 708-202-2413 708-202-2415 (fax)* [*virec@va.gov*](mailto:virec@va.gov)*.’*

*Please suggest appropriate updates if so desired, we’d like to reference a similar paper from Osborne et al. published by PlosOne on July 27^th^, 2020* [*https://journals.plos.org/plosone/article?id=10.1371/journal.pone.0236554*](https://journals.plos.org/plosone/article?id=10.1371/journal.pone.0236554)*, that outlined the same restrictions.*

*To clarify, for other VA researchers and their collaborators, data could be made accessible if working under an IRB approved protocol.*

## Reviewer 1

This study re-calibrated the labMT sentiment dictionary on 3.5M clinical notes describing 10,000 patients diagnosed with lung cancer at the Department of Veterans Affairs. This is an interesting research topic in oncology domain, could be helpful for patient care in practice in the future. Some of my concerns focus on the following points:

1. Your goal is to gauge if the Hedonometer, after re-calibration, can detect a signal indicating clinical trajectory of cancer care using medical record notes. Have you compared with the original Hedonometer before re-calibration? Is the original Hedonometer capable of detecting such a signal? Then what the advantage of the Hedonometer after re-calibration?

*We compared with the original Hedonometer before re-calibration and have included these results in the updated manuscript.*

*Briefly, the original Hedonometer was not capable of detecting a signal in the platelet comparison (p = 0.62), in contrast to the re-calibrated version. The re-calibrated Hedonometer also performed better than the original Hedonometer in regard to treatment analysis, as detailed in our response to item 5 below. We have updated the methods, results, and discussion to add comparison to the original Hedonometer.*

2. You calculated the sentiment score of notes and evaluated against a lab test and treatment. Is this evaluation based on the assumption that the sentiment from notes is associated with lab tests or treatments? Would it be possible that the sentiment comes from other events? How did you deal with this possibility?

*Our rationale for selecting platelet count as an objective measure is that it has been shown to be an indicator of how well a patient is feeling and has been linked extensively to cancer. The hypothesis is that if a patient is not feeling well, which could relate to an abnormal platelet count, this ‘unwell feeling’ is recorded in clinical notes, resulting in a drop in the sentiment score. However, we do acknowledge that the outcome of the validation of the Hedonometer on these two measures does not preclude the sentiment changes from being related to other events in patients' care. It merely shows presence of a measurable signal. We have added these points to the methods (under Methods – Platelet Count) and discussion.*

3. What is the rationale to determine the word frequency in your method: “obtaining the frequency of the unique words occurring in the note, looking up their score in the re-calibrated word list, and multiplying the frequency of the word by its score.”

*The rationale behind word score frequency is utilizing it to calculate the average score of a single note, this score is then further utilized in the analysis. We’ve updated the methods section accordingly with this.*

*In addition, we’ve made the code available for calculating note scores through Github, see:* [*https://github.com/delbers/SentimentAnalysisOfMedicalRecordNotes/blob/main/Code/score_note.py*](https://github.com/delbers/SentimentAnalysisOfMedicalRecordNotes/blob/main/Code/score_note.py)*.*

4. In the sentence: “All clinical notes one week before and one week after a platelet count test was performed were selected and individually scored.” Who scored these clinical notes? What is the inter annotator agreement?

*Average note scores are calculated utilizing the recalibrated Hedonometer Word Score methodology as described under 3. As this is an automated method, inter annotator agreement is not applicable. Though slightly different from IAA, but comparable, the standard deviation of individual re-scored words by the Subject Matter Experts is made available in Table S2. The calculation method has been more strongly referenced in the methods section.*

5. “According to SME’s in clinical lung cancer, this cyclical pattern can be explained due to patients’ weekly visits with their providers at which point they are asked about side-effects, bringing down the sentiment score of generated notes.” My question regarding this is if the dictionary before re-calibration can also produce the same result?

*The cyclical pattern was visible as well before recalibration; however it is not possible to find out what the oncology and healthcare specific words were that attributed to the cyclical pattern as highlighted in Fig 4, as these are not part of the original dictionary. Moreover, the average score was 0.3 points higher. This difference was attributed to the prevalence of missing clinical words in labMT, which were generally scored low by the SME’s. This information is present in the results section.*

6. What do you mean here ? “The re-calibration of the labMT sentiment dictionary for the oncology healthcare domain resulted in 200 re-scored words, which together cover 30% of clinical note text for the oncology domain”

*This references back to the first section of the Results, where we describe that the 200 words selected for inclusion in the oncology specific word dictionary, and scored by Subject Matter Experts, cover 30% of all words in the clinical notes in the lung oncology domain within the VA. Thus, if we had only used the original Hedonometer dictionary the coverage of words being used to calculate the average sentiment for a clinical note would have been (on average) 30% less. The discussion has been updated with this reference and with a reference to Fig 1.*

7. “Additionally, a consistent cyclic effect is visible when scoring notes in relation to cancer treatment timelines, showing a comparable cycle for all treatments.” Again, what is the result from the dictionary before re-calibration?

*Addressed in 5.*

8. What is your limitation in this study?

*A limitation of our re-calibration methodology is that only five SME's were included in the re-calibration, while a higher number would have improved its value. Identifying highly skilled SME's in the lung oncology domain with sufficient expertise is difficult and we are thankful for their given time.*

*Additionally, the outcome of the validation of the Hedonometer on these two measures does not preclude the sentiment changes from being related to other events in patients' care. Merely it shows presence of a measurable signal.*

*We like to emphasize that this is an explorative manuscript and is by no means exhaustive, future research should be done to understand better how and where the instrument can be utilized and what its limitations are in the oncology and healthcare domain. The finding that a signal appears present, detectable, and consistent is encouraging. These points have been added to the discussion.*

English errors:
As treatments might be given in combination or sequence, patients can be counted more then once.

*We thank the reviewer for noticing this mistake in English grammar and have updated the sentence as follows:*

*‘As treatments might be given in combination or sequence, patients can be counted multiple times’*
