# Supplementary material for: Sentiment analysis of medical record notes for lung cancer patients at the Department of Veterans Affairs
Source: PLoS One. 2023 Jan 25;18(1):e0280931. doi: 10.1371/journal.pone.0280931 (PMC9876289; doi:10.1371/journal.pone.0280931)
Supplement: Attachment — Submitted filename: Response to Reviewers.docx [file pone.0280931.s005.docx]

# Response to Reviewers (PONE-D-22-11193)

Based on comments from the reviewers the manuscript ‘Sentiment analysis of medical record notes for lung cancer patients at the Department of Veterans Affairs.’ has been adjusted. Our response can be found below.

## Reviewer 1

All comments have been addressed, no additional comments.

## Reviewer 2

Thank you for your efforts, please find below my comments for improvements and some suggestions

Title: looks good and reflects the methodology and aim of the research

Introduction:

Good explanation of sentiment analysis in a simple way

Part of sentiment analysis negotiation and that you agree with them better present in the discussions and limit the introduction for the currently available evidence and use of this type of analysis in healthcare systems. Also, you may add information about how the previous sentimental analysis helped and affected the healthcare systems. You have to use evidence with references.

You mentioned what you have done in the introduction, again this should be in the methodology and the discussion part. Try assessing where is the gap you aim to fill with your research and mention it in the introduction.

I would suggest that you paraphrase and clearly state your aim at the end of the introduction section.

*We have restructured the paper, in particular the introduction and discussion, as suggested.*

Methods:

In the selected data section, I suggest you clearly mention your inclusion and exclusion criteria.

*This has been updated in the Methods – Selected Data section with the addition of the VA’s Tumor Registry codes used to determine lung cancer. A reference has been added as well.*

Have you obtained Ethics Review Board? Please mention this in the methodology

*The Ethics Review Board (Institutional Review Board) is added including the number of the study, in accordance with PLOS ONE’s guidelines, under Methods – Selected Data.*

I suggest moving this “This resulted in a mean of 886 notes per patient per year and a standard 52 deviation of 2180.” To the results section.

*This has been moved to the Results*

Where is the reference for lines 58,59?

*The appropriate reference has been added.*

What systems, programs, or program languages have you used? Please mention that

*A new subheader ‘Software and Systems’ had been added to Methods.*

Please justify and explain lines 96-99 in more detail.

*This is further expanded upon in the Methods – Calculating Sentiment paragraph and includes a reference to the general practice of the Hedonometer, referencing the descriptive methodology paper by Dodds et al. (2011).*

What did you use to perform the statistical analysis?

*Python 3.8, specifically the scipy, statsmodels and scikit packages. This has been added to the Software and Systems paragraph.*

Results:

Line 152 is missing table number

*The table number has been added.*

I suggest that explaining the meaning of the results like in line 192 to be moved to the discussion section.

*This was moved to the Discussion.*

Discussion

I appreciate that you compared your data to other studies

Please elaborate more on the explanation of the result’s meaning and how it can affect the practice. Also, you may mention some of the limitations of your study. Furthermore, clear mention of what further studies need to be done in the future would be important and would enrich your manuscript.

*This has been elaborated upon and limitations have been added into the Discussion.*
